# PM2.5 Exposure in the Respiratory System Induces Distinct Inflammatory Signaling in the Lung and the Liver of Mice

**DOI:** 10.1155/2019/3486841

**Published:** 2019-12-01

**Authors:** Soi Jeong, Sang A Park, Inwon Park, Pilhan Kim, Nam Hoon Cho, Jin Won Hyun, Young-Min Hyun

**Affiliations:** ^1^Department of Anatomy, Yonsei University College of Medicine, Seoul, Republic of Korea; ^2^Brain Korea 21 PLUS Project for Medical Science, Yonsei University College of Medicine, Seoul, Republic of Korea; ^3^School of Medicine, CHA University, Seongnam, Republic of Korea; ^4^Department of Emergency Medicine, Seoul National University Bundang Hospital, Seongnam, Republic of Korea; ^5^Graduate School of Medical Science and Engineering, Korea Advanced Institute of Science and Technology, Daejeon, Republic of Korea; ^6^Graduate School of Nanoscience and Technology, Korea Advanced Institute of Science and Technology, Daejeon, Republic of Korea; ^7^KI for Health Science and Technology, Korea Advanced Institute of Science and Technology, Daejeon, Republic of Korea; ^8^Department of Pathology, Yonsei University College of Medicine, Seoul, Republic of Korea; ^9^Department of Biochemistry, School of Medicine, Jeju National University, Jeju, Republic of Korea

## Abstract

Fine particulate matter 2.5 (PM2.5) is a harmful air pollutant currently threatening public health. Although many studies have been performed on the general negative effects of PM2.5 in mice and humans, the migration patterns of various immune cells in response to PM2.5 exposure remain unclear. In this study, we aimed to investigate the immune cell migratory response in the lung and the liver of intratracheally PM2.5-inoculated mice. To investigate the migration trajectory of immune cells in the lung and the liver tissues of mice, we employed microscopic tools including two-photon intravital imaging, histological analysis, and transmission electron microscopy. Our data from two-photon intravital imaging showed that there was no significant difference in the number of infiltrated neutrophils in the lung and the liver of PM2.5-treated mice, compared to the nontreated condition. However, from the histological analysis and the transmission electron microscopy after vascular perfusion to remove intravascular leukocytes, we observed that some leukocytes were frequently observed in the lung and the liver of PM2.5-treated mice. Interestingly, quantification of leukocyte population using flow cytometry showed significant increase of neutrophils and macrophages in the lung, but not much in the liver, 24 h post-PM2.5 treatment. These data imply that two-photon intravital imaging of the lung and the liver actually visualized neutrophils, which were adherent to the luminal side of the vasculature. We then conducted mRNA microarray analysis to further observe how PM2.5 affects gene expression patterns in the lung and the liver. PM2.5 treatment changed the mRNA expression associated with the IL-17 signaling pathway in the lung and changed the mRNA expression associated with metabolic pathways in the liver. In summary, these results suggest that the immune response in the lung is distinctly regulated from that in the liver under acute PM2.5-induced inflammation and that these organs consequently are regulated via distinct signaling pathways.

## 1. Introduction

Particulate matter (PM2.5) has been a focus of interest among various artificial pollutants in recent years worldwide because of its biohazardous effects. Unlike naturally occurring “dust,” PM mostly comprises chemicals that are emitted from diesel, construction factories, and power plants [1]. PM can be classified into two groups according to size: PM10 and PM2.5. PM10 refers to particulate matter that has a diameter smaller than 10 *μ*m, whereas PM2.5 has a diameter smaller than 2.5 *μ*m. Although the components of PM differ depending on its sources, such as heavy metals and acidic substances, it is mainly composed of black carbon. PM can be inhaled into the trachea and the lung as it is extremely small and easily deposited into the alveoli and even the bloodstream, resulting in an inflammatory response. Therefore, it is assumed that this inflammation may not only be the cause of respiratory disorders but could also trigger the permanent damage of various organs. Exposure to PM2.5 also leads to poor reproductive ability [2] and/or decreased brain functions [3].

The lung is a critical organ that manages the exchange of oxygen and carbon dioxide in the bloodstream. Because of the lung's direct contact with air, this organ inevitably confronts many pathogens, ranging from dust to viruses. When trachea and lung tissues are exposed to artificial contaminants, leukocytes secrete cytokines and chemokines that cause acute inflammation [4], and PM can induce and intensify the secretion of these signaling molecules. Therefore, PM affects the pulmonary pathways and aggravates diseases such as chronic obstructive pulmonary disease [5, 6], pneumonia [7], and asthma [8]. The effects of PM2.5 on the lung have been studied over the past few decades, but studies reporting those on the liver are fairly rare. The liver detoxifies hazardous substances in the body and purifies the blood [9, 10]. However, because the liver is known as a “silent organ,” it is difficult to detect the symptoms of inflammation when individuals are exposed to PM2.5. Here, we examined whether PM2.5 affects the lung and the liver by causing an inflammation in a PM-exposed mouse model. In addition, microarray analysis was conducted to investigate the signaling pathways of the lung and the liver.

## 2. Methods

### 2.1. Mice

C57BL/6 mice and lysozyme M-green fluorescent protein- (LysM-GFP-) expressing mice [11], aged 6 to 10 weeks, were used. C57BL/6 mice were purchased from Orient Bio (Orient Bio Inc., Seongnam, Korea) and were housed at the Avison Biomedical Research Center (ABMRC) of Yonsei University College of Medicine. The mice were divided into three groups according to treatment and time after PM2.5 exposure: (1) control group treated with deionized distilled water, (2) mice assessed 24 h post PM injection, and (3) mice assessed 48 h post PM treatment. All animal studies were approved by the Institutional Animal Care and Use Committee of the Yonsei University College of Medicine (IACUC No. 2019-0097).

### 2.2. PM2.5 Treatment

PM2.5 was purchased from Sigma-Aldrich (St. Louis, MO, USA; product number: NIST 1650b). It was dissolved in 100% dimethyl sulfoxide (DMSO) and washed with deionized distilled water three times. PM2.5 in distilled water was sonicated for 3 min to minimize aggregation. For the injection of PM2.5 into the mouse lung, catheters were directly intubated into the mouse airways and 25 *μ*l of solution containing 200 *μ*g PM2.5 was introduced via intratracheal injection [12]. After PM injection, mice were allowed to rest for at least 18 h. The effects of PM2.5 were investigated 24 h and 48 h after injection.

### 2.3. Two-Photon Intravital Imaging of Mouse Lung and Liver

Intravital imaging was conducted via two-photon microscopy to observe the neutrophils in the lung and the liver of mice. An LSM 7MP microscope (Carl Zeiss Microscopy, Jena, Germany) equipped with a water-dipping lens was used. The instrument was equipped with a two-photon laser (690-1040 nm, MaiTai HD DeepSee tunable laser) and was set to detect Dextran-Texas red and LysM-GFP at a wavelength of 880 nm. ZEN 2010 software (Carl Zeiss) was used to analyze two-photon microscopy images. For the intravital imaging of lung tissues, thoracotomy was performed according to methods previously described and slightly modified [13, 14]. Mice were anesthetized with a Zoletil-Rompun mixture diluted 1 : 10 (*v* : *v*) in phosphate-buffered saline (PBS). Dextran-Texas red (70 kDa, Thermo Fisher Scientific, Waltham, MA, USA) was injected via retro-orbital method to visualize mouse blood flow. Mouse intubation using a 20G × 1.16 intravenous catheter was conducted prior to thoracotomy. To prevent the detachment of the cannula from the ventilator, the connection area was fixed with tape and subsequently sutured to the mouth of each mouse. Skin and fat tissue were carefully removed in order to expose the ribcage. The ribs were removed with surgical scissors, and the lung chamber was fixed prior to imaging. For intravital imaging of the liver, mouse anesthesia and blood flow labeling were performed using the method described above. The mouse's abdominal hair was removed with a hair removal cream before surgery. The premarked incision area (from the xiphoid process to the very end of the right rib) was cut using forceps and scissors. The left medial lobe of the liver was removed from the abdominal cavity and then excised using cotton swabs. The mouse was positioned on the imaging chamber in a side-on position. The mouse's liver was carefully attached on the silicon bed for fixation. The cover glass was placed on the fixed liver and imaging was performed [15].

### 2.4. Imaging Data Analysis

Volocity (PerkinElmer, Waltham, MA, USA) and the open-source software FIJI (NIH) were used for leukocyte counting and data analysis. Prism software (GraphPad, La Jolla, CA, USA) was used to present the mean values of cells in each group under different experimental conditions.

### 2.5. Hematoxylin and Eosin (H&E) Staining

The left lung and the medial lobe of each liver were removed from mice and then perfused with 1x PBS buffer. Extracted organs were fixed for 4 days in 4% formalin. The fixed tissue was embedded in paraffin via dehydration and clearing. Paraffin blocks were cut into 5 *μ*m sections using a microtome. Before deparaffinization and rehydration, tissue sections were stained with H&E and visualized under a microscope using a ×200 objective lens (Olympus BX51 microscope, Olympus, Tokyo, Japan).

### 2.6. Transmission Electron Microscopy (TEM)

The left lung and the medial lobe of each liver were perfused with 1x PBS buffer. Samples were then fixed with Karnovsky's fixative (2% glutaraldehyde, 2% paraformaldehyde, 0.5% CaCl_2_) at pH 7.4 for more than 12 h, followed by washing and secondary fixing of the sample. Samples were dehydrated in alcohol and placed in propylene oxide. Epon 812 (methyl nadic anhydride (MNA), dodecenyl succinic anhydride (DDSA), 2,4,6-Tris-(dimethylaminomethyl)phenol (DMP30)) and propylene oxide were mixed at a ratio of 1 : 1 (*v* : *v*), and samples were rested overnight. Samples were then baked in an electron microscopy (EM) oven at 35°C for 6 h, followed by 12 h at 45°C, and finally 24 h at 60°C. Blocks were trimmed using an ultramicrotome. Tissues were sectioned to a thickness of 200-250 nm, stained with 1% toluidine blue, and retrimmed at the sites of observation, and ultrathin sections (80 nm) were prepared. The sections were placed on a copper grid and double-stained with uranyl acetate (6%) and lead citrate. All sections were visualized via TEM (JEM-1011, JEOL, Tokyo, Japan) at an acceleration voltage of 80 kV.

### 2.7. Flow Cytometry Analysis

The PM2.5 was administered to the mouse under the same conditions as described above. Lungs and livers of mice were chopped for the preparation of single cells. Lung tissue was incubated for 1 h at 37°C, 150 rpm in 5 ml HBSS containing 100 *μ*l of 5% collagenase type IV, 150 *μ*l of 0.1 M CaCl_2_, and 25 *μ*l of DNase I (1 mg/ml). The digested lung tissue was filtered into 70 *μ*m cell strainer. Single cells were obtained from the lung by centrifuging the filtered solution at 1,500 rpm at 4°C for 3 min. Liver tissue was incubated in 10 ml HBSS containing 500 *μ*l of 5% collagenase type IV and 25 *μ*l of DNase I (1 mg/ml) for 30 min at 37°C, 150 rpm. The digested liver tissue was filtered into a 70 *μ*m cell strainer. Single cells were obtained from the liver by centrifuging the filtered solution at 400 g at 4°C for 10 min. For flow cytometry analysis of single cells from the lung and the liver, T cells were stained with anti-CD3-APC/Cy7 (BioLegend) and macrophages were stained with anti-F4/80-PE (BioLegend) and neutrophils were stained with anti-Ly6G-FITC (BioLegend). Fluorescence was measured using LSRII or LSRFortessa (BD Biosciences). The data were analyzed using FlowJo software.

### 2.8. Microarray and Statistical Analysis

Total RNA was isolated from each PM2.5-treated organ according to the manufacturer's instructions (Macrogen, Seoul, South Korea). The Affymetrix whole-transcript expression array process was performed according to the manufacturer's instructions (GeneChip Whole-Transcript PLUS Reagent Kit, Affymetrix, Santa Clara, CA, USA). Biotin was tagged with terminal deoxynucleotidyl transferase (TdT) using the GeneChip WT Terminal labeling kit. The DNA target tagging was hybridized to an Affymetrix GeneChip Mouse 2.0 ST array. Results were subjected to robust multiarray average (RMA) analysis at the genetic level, and differential expression of gene (DEG) analysis was performed. A comparative analysis of the test samples and the control samples was carried out using fold change. Gene ontology (http://geneontology.org/) and the Kyoto Encyclopedia of Genes and Genomes (KEGG) (https://kegg.jp) were used to perform gene-enrichment and functional annotation analysis for a significant probe list. Microarray data analysis was performed by Macrogen. All statistical testing and visualization of differentially expressed genes were performed using R statistical language v. 3.1.2. GSEA was performed using normalized signal of microarray to quantify a number of gene sets with statistically significant and concordant differences.

### 2.9. Quantitative Real-Time PCR (qPCR)

mRNA was prepared from each organ using the TRIzol™ Reagent following manufacturer's instructions (Invitrogen). The cDNA was synthesized using AccuPower RT PreMix following manufacturer's instructions (Bioneer). SYBR Green PCR Master Mix (Applied Biosystems) was used for qPCR using the StepOnePlus Real-Time PCR system (Applied Biosystems). For qPCR, we used CCL5 primers: the forward one was CCCTCACCATCATCCTCACT and the reverse one was CCTTCGAGTGACAAACACGA. Also, for qPCR of *β*-actin, the forward one was CATGTTTGAGACCTTCAACACCCC and the reverse one was GCCATCTCCTGCTCGAAGTCTAG. qPCR condition was 95°C (3 min), followed by 40 cycles of 95°C (5 sec), 60°C (10 sec), and 72°C (10 sec). We used *β*-actin as a normalization control.

## 3. Results

### 3.1. PM2.5 Did Not Increase Neutrophil Number in the Lung and the Liver of Live Mice

**Figure 1:**
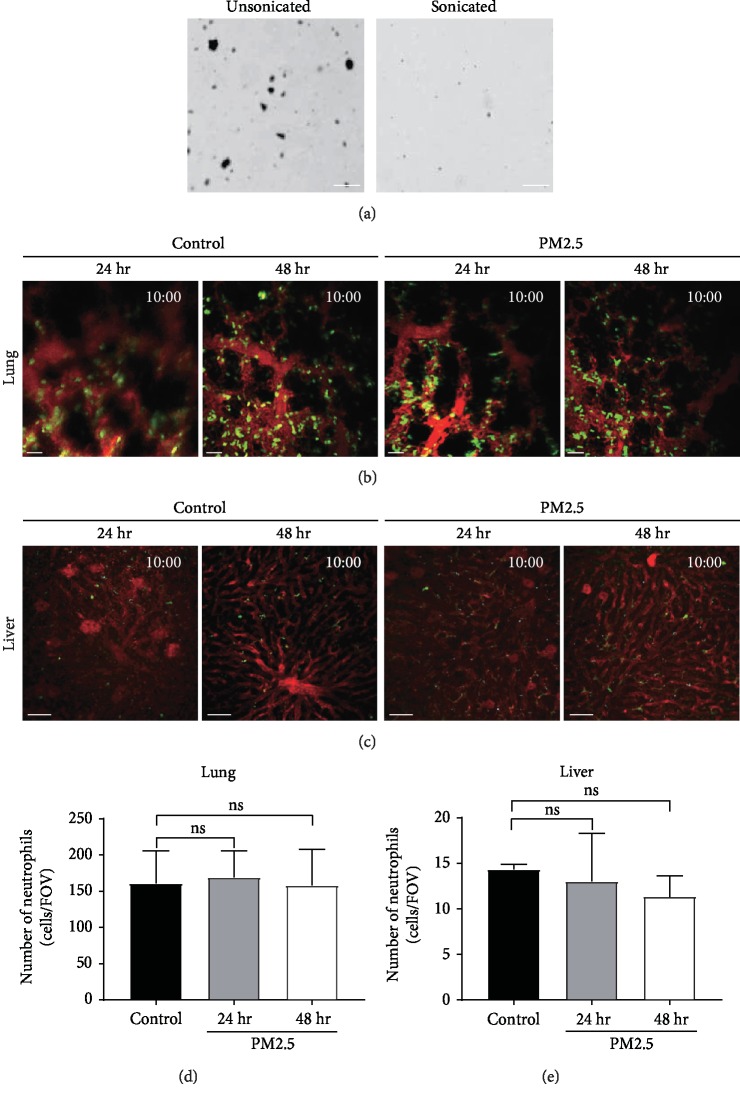
Two-photon intravital imaging of neutrophils in the lung and the liver of mice exposed to particulate matter 2.5. (a) Images show the difference between PM2.5 before and after sonication. Scale bar, 10 *μ*m. LysM-GFP mice were treated with PM2.5, and the number of neutrophils was recorded 24 h and 48 h after treatment via two-photon intravital imaging of mouse lung (b) and liver (c), respectively. See Videos [Supplementary-material supplementary-material-1], [Supplementary-material supplementary-material-1], [Supplementary-material supplementary-material-1], and [Supplementary-material supplementary-material-1]. The blood vessel was stained with Dextran-Texas red. Scale bar, 50 *μ*m. The number of neutrophils at 10 min was counted from two-photon imaging of mouse lung (d) and liver (e), respectively. There was no significant increase in the neutrophil number in the lung and liver tissues compared to that in the control group.

PM2.5 is composed of water-soluble and insoluble components [16]. To introduce PM2.5 directly into the mouse's trachea, it was sonicated in deionized distilled water so that the small particles were well mixed. The PM2.5 particle size was measured before and after sonication using a microscope (Figure 1(a)). After sonication for 3 min prior to intratracheal injection, aggregated PM2.5 particles were evenly distributed and clusters were undetectable. Two-photon intravital imaging was performed with LysM-GFP mice, and numerical changes in the migratory neutrophils in each organ after PM2.5 treatment were measured. To investigate the effect of PM2.5 treatment on neutrophil migration, mice were divided into three groups: (1) a control group (receiving intratracheal injection of deionized distilled water), (2) a group assessed 24 h post-PM2.5 treatment, and (3) a group assessed 48 h post-PM2.5 treatment. Under these conditions, the lung and the liver were independently imaged (Figures 1(b) and 1(c) and Videos [Supplementary-material supplementary-material-1], [Supplementary-material supplementary-material-1], [Supplementary-material supplementary-material-1] and [Supplementary-material supplementary-material-1]). Cell numbers were recorded at 10 min during intravital imaging. According to the two-photon intravital imaging analysis of both lung and liver tissues, there was no significant difference in the number of neutrophils of the three groups in these two organs (Figures 1(d) and 1(e)). In addition, neutrophil shape did not change after PM2.5 administration. Since two-photon intravital imaging was performed using live mice without vascular perfusion, we supposed that neutrophils were counted from the tissue and the blood vessel of these organs. Therefore, these data imply that respiratory system exposure to PM2.5 does not change the overall number of neutrophils in the lung and the liver, when counted on the intact tissue without vascular perfusion.

### 3.2. Leukocytes Were Frequently Observed in the Lung and the Liver Tissues of PM2.5-Treated Mice

**Figure 2:**
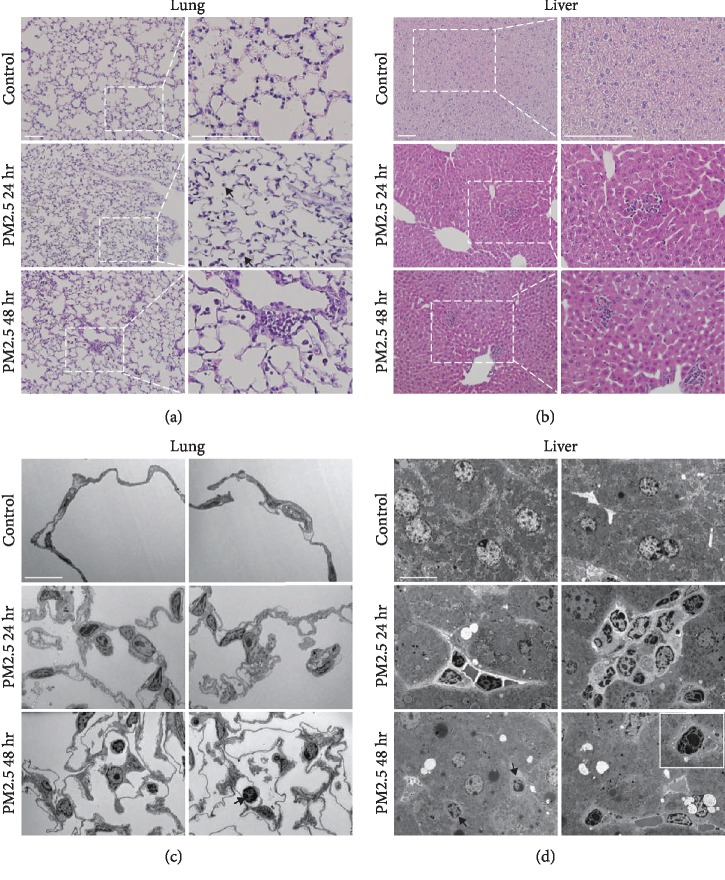
Leukocytes in hematoxylin and eosin- (H&E-) stained tissue sections and transmission electron microscopy (TEM) images after PM2.5 exposure. (a, b) The histopathological structures of lung and liver tissues were confirmed by H&E staining after PM2.5 treatment. In the PM2.5-treated group, lymphocytes were observed in both organs. Scale bar, 20 *μ*m. (c) TEM images of the lung revealed that the number of lymphocytes in the alveolar sacs after PM2.5 treatment increased. (d) Liver imaging showed the formation of lymphocyte clusters and indicated that an inflammatory reaction had occurred. An eosinophil was also observed (white box). Black arrows indicate lymphocytes in the bottom panels of (c) and (d). Scale bar, 10 *μ*m.

**Figure 3:**
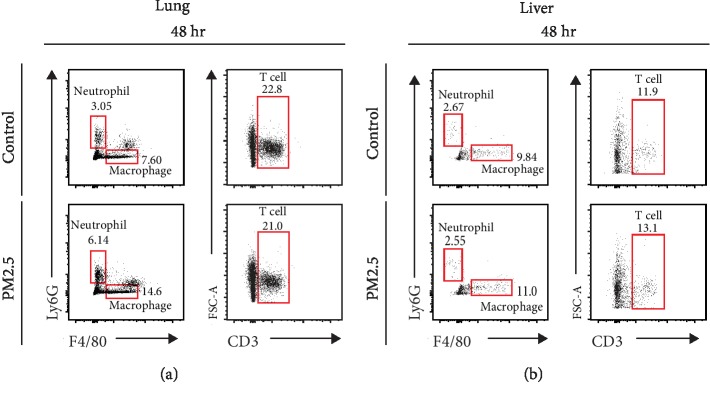
Quantitative analysis of leukocyte infiltration in the lung and the liver after PM2.5 treatment. Neutrophil, macrophage, and T cells were counted by flow cytometry in the 48 h post-PM2.5 treatment group. A representative of three repeated flow cytometry plots showed gating leukocytes in the lung (a) and the liver (b).

The movement of leukocytes into tissues indicates the occurrence of an inflammatory response in the tissue [17]. As we did not observe the number of neutrophils significantly increase in two-photon intravital imaging of LysM-GFP mice, we performed H&E staining to investigate various immune cell infiltration in the fixed tissues of the lung and the liver of mice after vascular perfusion. In the lung, the control group showed uniformity of the alveoli sac structure where gas exchange occurs. However, both PM2.5-treated groups showed relatively dense alveoli sac structures compared to the control group (Figure 2(a)). In addition, lymphocytes were observed outside the alveolar sac of the 24 h post-PM2.5 treatment group, and clusters of lymphocytes and neutrophils were observed in the 48 h group (Figure 2(a)). In the liver, hepatocytes were tightly clustered with one another in the control group. However, in both PM2.5-treated groups, gaps composed of lymphocytes were formed between the hepatocytes (Figure 2(b)). Furthermore, TEM was performed to further finely investigate immune cell location and structural alteration of both organs. In the lung, leukocytes were frequently found in the alveolar epithelium forming the sac or outside the sac (Figure 2(c)). In the liver, lymphocyte clusters were detected after PM2.5 treatment (Figure 2(d)). Arrows in Figures 2(c) and 2(d) indicated lymphocytes. Eosinophils were also confirmed in TEM data, though not many (the bottom right panel in Figure 2(d)). Eosinophils are cells that typically cause inflammation as part of the allergic reaction [18]. In summary, leukocyte infiltration in the lung and the liver tissues lasted for a minimum of 2 days post-PM2.5 treatment and caused an inflammatory reaction in this area. As the immune cells were identified in the data above, flow cytometry was further performed specifically to determine what kinds of immune cells were infiltrated only in the lung and the liver tissues excluding immune cells in the blood vessels by vascular perfusion. Neutrophils, macrophages, and T cells were counted in both lung and liver using flow cytometry. Compared to the control group, the number of neutrophils and macrophages in the lung was increased in the 48 h group post-PM2.5 treatment (Figure 3(a)). In the liver, no immune cells were increased at 48 h after PM2.5 treatment (Figure 3(b)). Thus, we observed that different immune cells were infiltrated depending on the organs after PM2.5 stimulation.

### 3.3. Distinct Signaling Occurred in the Lung and Liver Tissues following PM2.5 Treatment

**Figure 4:**
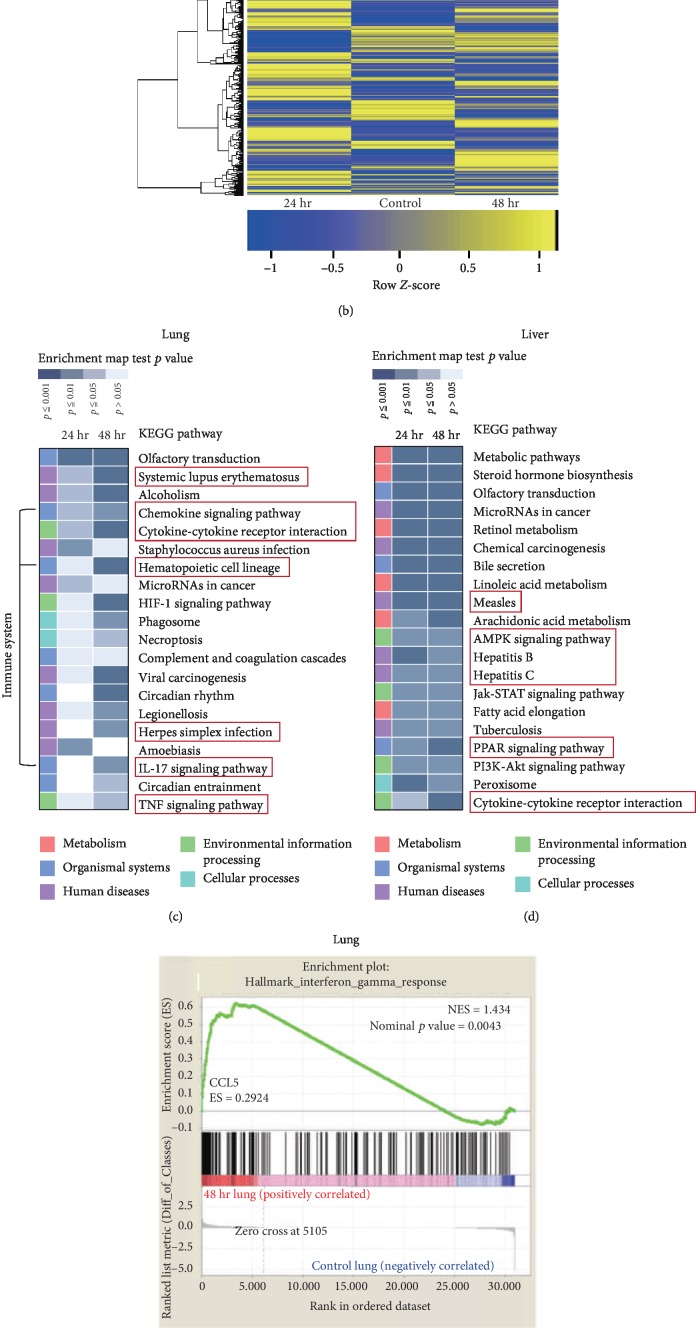
Comparison of mRNA expression pattern in the lung and the liver after PM2.5 treatment. (a, b) Microarray cluster images comparing the PM2.5 treatment groups and control group of the lung and the liver, respectively. (c, d) The 20 most significant Kyoto Encyclopedia Gene and Genome (KEGG) pathways with increased mRNA expression after PM2.5 treatment were shown for each organ. The red box showed the pathways associated with the immune system, cytokines, and human disease. (e, f) The GSEA results of the IFN-*γ* response pathway were shown; the common pathway in the lung and the liver of mice assessed 48 h post-PM2.5 treatment was significantly increased with an NES of 1.792 and 1.434, respectively. (g) Quantitative real-time PCR analysis (2^−ΔΔCt^ method) of CCL5 gene expression was shown in the control group and the 48 h post-PM2.5 treatment group of the lung and the liver. Data was presented as mean ± SEM (*n* = 5).

We performed microarray to determine whether there is any change of mRNA expression related to leukocyte infiltration in response to PM2.5 treatment in the lung and the liver. Microarray cluster imaging showed different levels of mRNA in PM2.5-treated organs compared to those of the control group. The number of each probe represented the number of mRNAs on the microarray chip, with the difference in number of linked probes indicating the gaps in mRNA expression. In the lung tissues, a change in mRNA expression 48 h post-PM2.5 treatment was more frequent than that in the 24 h group (Figure 4(a)). Conversely, in the liver tissues, the mRNA expression 24 h after PM2.5 treatment was more significantly changed compared to that in the 48 h group (Figure 4(b)). The enrichment map demonstrated that the changes in mRNA expression are related to specific signaling pathways. In lung tissues, the expression of the immune system-related pathways (red box) or cytokines changed in the PM2.5-treated group compared to the control group (Figure 4(c)). In this map, the IL-17 signaling pathway was significantly altered in the lung 48 h after PM2.5 treatment. IL-17 is a proinflammatory cytokine that is expressed by helper T cells. The changes in IL-17 may be associated with lymphocyte infiltration in PM2.5-treated lung tissues. In liver tissues, the pathway associated with metabolism was found to be more significantly affected (Figure 4(d)). This suggests that PM2.5 exposure may affect metabolic diseases associated with lipid accumulation, oxidative stress, and insulin resistance in the liver as suggested by previous findings [19]. This may be due to a specific response associated with metabolism occurring in the liver in the presence of PM2.5.

As microarray data showed that a particular set of genes was significantly changed, additional GSEA was performed to explore which genes had increased. To focus on the immune response with a common pathway in both organs, a pathway with a high NES value and a *p* value below 0.05 was selected (nominal *p* value of the lung: 0.0043, nominal *p* value of the liver: 0). There was no common pathway selected in both 24 h groups. However, we found that the CCL5 gene set of the interferon gamma (IFN-*γ*) response pathway of the Hallmark gene set was significantly increased in the 48 h group of the lung and the liver (Figures 4(e) and 4(f)). IFN-*γ* was secreted by NK cells and T cells. As originally defined as a product with direct antiviral activity, IFN-*γ* modulates various aspects of the immune response, including the coordination of leukocyte-endothelium interactions, cell proliferation, and cell death [20]. Also, CCL5 mainly acts at inflammatory sites and is involved in recruiting various leukocytes including T cells, macrophages, eosinophils, and basophils. CCL5 interacts with specific cytokines such as IL-2 and IFN-*γ*, which are released by T cells [21]. Based on our GSEA, we focused on CCL5, which was shown to be commonly increased in the liver and the lung of the 48 h group post-PM2.5 treatment. qPCR analyses were performed using the delta cycle threshold method. The mRNA level was increased in the 48 h post-PM2.5 treatment group in both lung and liver, compared to the control group. The graph showed mRNA expression of CCL5 in the lung at 2.44-fold higher than that of the control group. In the liver, the mRNA expression of CCL5 was 1.03-fold higher than that of the control (Figure 4(g)).

## 4. Discussion

PM2.5 can reach almost all organs in the body via the bloodstream because of its extremely small particle size [22]. It was reported that high PM2.5 concentration in the air leads to increased cardiac and respiratory diseases [23]. The lung is an organ in which PM2.5 can be inhaled directly through the trachea, whereas the liver does not make direct contact with PM2.5. The liver was chosen as a comparative organ to investigate the effect of inhaled PM2.5 on the lung, because it plays a role as a detoxifying and symptomless organ at an early stage of damage [24]. Our comparison study of these two organs indicated that PM2.5 introduced through the respiratory tract can cause inflammation in the secondary organs other than the respiratory system via exposure through the bloodstream. Using two-photon microscopy, the specific structures of each organ, including the inner anatomy of the lung (alveoli and capillaries) and the liver, and the morphology of neutrophils in a mouse model were successfully monitored in a time-lapse manner. It is an invasive surgery to exteriorize the lung or the liver from live animal for intravital imaging. The surgery process may induce an inflammatory environment to the exteriorized organ tissue. Therefore, it is very important to minimize autonomous inflammatory condition in the organ tissue. To investigate the inflammatory effect of PM2.5 treatment to mice, we have been careful in performing the surgery with minimal autonomous inflammation with a strict control condition. We also conducted histological analyses with H&E staining and ultrastructural analyses with TEM as well as flow cytometry. In H&E staining and TEM imaging, we were able to observe leukocytes in both lung and liver tissues after PM2.5 treatment. The flow cytometry data showed that neutrophils were significantly increased in the lung within 48 h after PM2.5 treatment. Although this result was not in accordance with two-photon intravital imaging data detecting immune cells in both the tissue and the blood vessels, this still suggests an increase in neutrophil infiltration to the tissue.

The expression of the IL-17 pathway was changed in the enrichment map of the microarray, particularly, of the lung tissues. IL-17 expression was more significantly changed 48 h after PM2.5 treatment compared to that after 24 h in the lung tissues. When the lung is damaged, IL-17 is rapidly produced by pulmonary innate lymphoid cells (ILCs), which may explain our results as ILCs contribute greatly to the enhancement of early immunopathology [25]. The expression of pathways related to metabolism changed in the liver treated with PM2.5; more specifically, an increase in metabolism-associated liver function was observed [26]. As the liver has more than 500 distinct roles and functions, it is infeasible to discern which mechanism is directly affected by PM2.5. The pathway directly associated with the immune system has not been significantly changed. However, from GSEA, we could find that CCL5 gene expression increased, which is associated with the immune response.

Our data showed no significant change in neutrophil numbers, although these innate immune cells are known to infiltrate under inflammatory conditions. This may be attributed to the fact that the PM2.5 we used in this experiment differs from that in actual atmospheric conditions. PM2.5 in the atmosphere is mainly composed of a carbon backbone and additionally mixed with various materials such as SO_4_^2−^, NO_3_^−^, NH_4_^+^, organic matter, and crustal matter [27]. However, the PM2.5 used in our experiment is purely composed of diesel particulate materials with a simple carbon backbone. Most PM2.5 studies focus on the onset of chronic illness through long-term inhalation [28–31]. Here, we have conducted experiments on acute inflammatory responses within 48 h after PM2.5 treatment, which may hardly induce neutrophil infiltration. However, as far as we know, this study would be the first trial to investigate the migration pattern of each immune cell population using intravital imaging and verify distinct inflammatory signaling pathways in the lung and the liver of PM2.5-treated mice. Identification of distinct pathways in the lung and the liver tissues through microarray data suggests that the possibility of different treatment methods would be applied to patients suffering from diseases related to PM2.5 exposure, depending on their symptoms. Further studies on long-term exposure to PM2.5 may also provide in-depth information regarding the various pathways associated with prolonged inflammation.

## Data Availability

All data used to support the findings of this study are included within the article.

## Supplementary Materials

Supplementary 1 Supplementary Video 1: two-photon intravital imaging of the lung in LysM-GFP mice (a control group). The left side and the right side were taken 24 h and 48 h, respectively, post deionized distilled water treatment. Neutrophils were visualized in green in the mice. Blood vessels were visualized with i.v.-injected Dextran-Texas red. Scale bar: 50 *μ*m.

Supplementary 2 Supplementary Video 2: two-photon intravital imaging of the lung in LysM-GFP mice (PM2.5-treated group). The left side and the right side were taken 24 h and 48 h, respectively, post-PM2.5 treatment. Neutrophils were visualized in green in the mice. Blood vessels were visualized with i.v.-injected Dextran-Texas red. Scale bar: 50 *μ*m.

Supplementary 3 Supplementary Video 3: two-photon intravital imaging of the liver in LysM-GFP mice (a control group). The left side and the right side were taken 24 h and 48 h, respectively, post deionized distilled water treatment. Neutrophils were visualized in green in the mice. Blood vessels were visualized with i.v.-injected Dextran-Texas red. Scale bar: 50 *μ*m.

Supplementary 4 Supplementary Video 4: two-photon intravital imaging of the liver in LysM-GFP mice (PM2.5-treated group). The left side and the right side were taken 24 h and 48 h, respectively, post-PM2.5 treatment. Neutrophils were visualized in green in the mice. Blood vessels were visualized with i.v.-injected Dextran-Texas red. Scale bar: 50 *μ*m.
